# Validation of a Novel Traditional Chinese Medicine Pulse Diagnostic Model Using an Artificial Neural Network

**DOI:** 10.1155/2012/685094

**Published:** 2011-09-13

**Authors:** Anson Chui Yan Tang, Joanne Wai Yee Chung, Thomas Kwok Shing Wong

**Affiliations:** ^1^School of Nursing, Caritas Medical Centre, Hong Kong; ^2^Department of Health and Physical Education, Hong Kong Institute of Education, Hong Kong; ^3^Tung Wah College, Hong Kong; ^4^School of Nursing, The Hong Kong Polytechnic University, Hong Kong

## Abstract

In view of lacking a quantifiable traditional Chinese medicine (TCM) pulse diagnostic model, a novel TCM pulse diagnostic model was introduced to quantify the pulse diagnosis. Content validation was performed with a panel of TCM doctors. Criterion validation was tested with essential hypertension. The gold standard was brachial blood pressure measured by a sphygmomanometer. Two hundred and sixty subjects were recruited (139 in the normotensive group and 121 in the hypertensive group). A TCM doctor palpated pulses at left and right cun, guan, and chi points, and quantified pulse qualities according to eight elements (depth, rate, regularity, width, length, smoothness, stiffness, and strength) on a visual analog scale. An artificial neural network was used to develop a pulse diagnostic model differentiating essential hypertension from normotension. Accuracy, specificity, and sensitivity were compared among various diagnostic models. About 80% accuracy was attained among all models. Their specificity and sensitivity varied, ranging from 70% to nearly 90%. It suggested that the novel TCM pulse diagnostic model was valid in terms of its content and diagnostic ability.

## 1. Introduction

**Figure 1:**
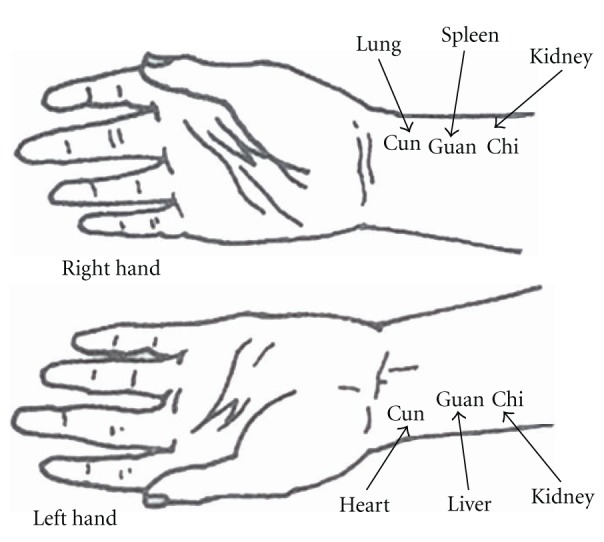
The six locations and their corresponding organs [13].

Traditional Chinese medicine (TCM) pulse diagnosis is one of the major clinical diagnostic methods in TCM. It has been used by TCM doctors to assess patients' health conditions for several thousand years. A TCM doctor palpates six locations, three on each wrist, with the three points called “cun”, “guan”, and “chi” [1, 2], and describes pulses in terms of various characteristics. By comparing the pulses at left and right cun, guan, and chi, the health status of individual organs and of the whole body can be determined [1–3]. As specified in the ancient texts, the Nan Jing [1] and Mai Jing [2], the heart, liver, and kidneys are assessed at the left cun, guan, and chi, respectively, while the lungs, spleen, and kidneys are assessed at the right cun, guan, and chi, respectively (Figure 1).

Numerous pulse qualities are documented in ancient Chinese medical texts. For example, the Nei Jing describes over 30 types [4], and the Mai Jing documents 24 types [2]. The most common 28 TCM pulse qualities were used in clinical practice come from the references Bin Hu Mai Xue [5] and Zhen Jia Zheng Yan [6]. These qualities are floating, sunken, slow, rapid, surging, fine, vacuous, replete, long, short, slippery, rough, string-like, tight, soggy, moderate, faint, weak, dissipated, hollow, drumskin, firm, hidden, stirred, intermittent, bound, skipping, and racing. These pulse qualities are largely described qualitatively and are not clearly defined. For instance, the slippery is compared to “beads rolling” and the string-like is like pressing the string of a musical instrument [5]. The magnitude of a pulse quality is, similarly, not precisely defined. The difference between a fine pulse and a faint pulse is that the former is a “little bit stronger” than the latter [7]. “A little” does not precisely determine what differentiates fine from faint. Thus, descriptions of pulse are subject to the interpretation of individual doctors, and this lack of quantitative standardization undoubtedly contributes to the low inter- and intrarater reliability among TCM doctors [8–10]. King et al. [11] tried to quantify TCM pulse diagnosis but did not reflect pulse qualities adequately for several reasons. First, the six items included in their scale-depth, width, force, relative force, rhythm, and pulse occlusion are not widely accepted as core items in TCM pulse diagnosis. Second, their scale was an ordinal scale anchored with descriptors to measure the items. For example, depth was measured at three levels: superficial, middle, and deep. However, an ordinal scale is not a sufficiently sensitive measure, as there are an insufficient number of available response categories to rate the items [12], and the words used to describe each ordinal level are not universal. Further, as the items have not been well quantified, using an ordinal scale would not reflect the actual sensation perceived by a TCM doctor.

To address these issues, the research team recontextualized TCM pulse diagnosis in an explicit and quantifiable way. This is significant because an effective treatment regimen relies on accurate clinical diagnostic data which can only be obtained by using an assessment method with high accuracy. 

To recontextualize TCM pulse diagnosis, several issues must be addressed. First, the qualitative descriptions of pulse qualities must be quantified and comparable. Second, the integrity of the pulse diagnosis must be preserved. Third, it must possess adequate validity in assessing health status. We aim at introducing a novel TCM pulse diagnostic model to recontextualize TCM pulse diagnosis and validate the model with essential hypertension. Essential hypertension is selected in this study because much evidence shows an association between TCM pulse qualities and essential hypertension [14–22]. The null hypothesis is that the TCM pulse diagnostic model is not accurate in essential hypertension diagnosis.

### 1.1. TCM Pulse Diagnostic Model

Research papers and reviews found in several databases, including Chinese Medical Current Contents (1994–2011), CBMdisc (1980–2011), and CAJ Full-text Database, concur that TCM pulse quality should be described by eight elements with various intensities. The eight elements are depth, rate, regularity, width, length, smoothness, stiffness, and strength [7, 23–27]. Rate is the number of beats per breath. The definition of regularity is similar to that in Western medicine, in that it describes the rhythm of a pulse [14]. Depth is defined as the vertical position of a pulse [14]. Width and length describe the shape of a pulse, where width is defined as the intensity of a pulsation and length is defined as the range in which the pulsation can be sensed across the cun, guan, and chi [7]. Smoothness is defined as the slickness of a pulse, stiffness is defined as the sensation of arterial elasticity, and strength is defined as the change in forcefulness of a pulse in response to a change of applied pressure [14]. The diagnostic model proposed in this paper is based on these eight elements. 

**Figure 2:**
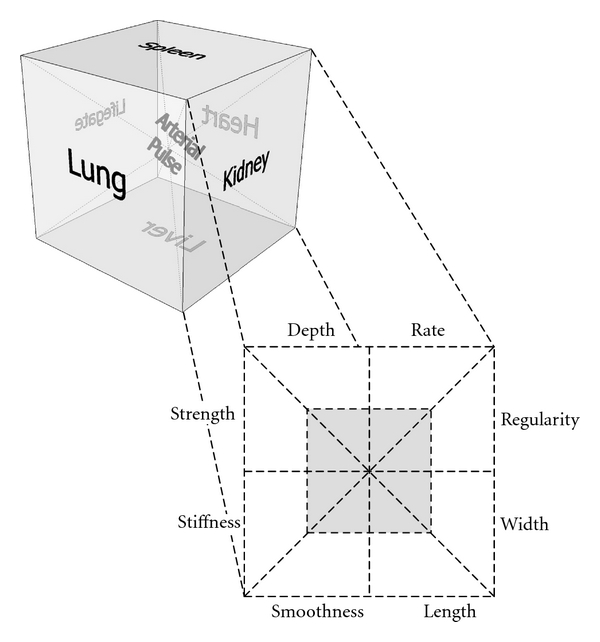
TCM pulse diagnostic model.

As illustrated in Figure 2, a die was adopted to represent the intertwining and cascading relationship among an arterial pulse and the eight elements at the six locations and the health status. The dotted line in Figure 2 links the six pyramids together to symbolize the interchanging and dynamic relationship among the organs. Yin and Yang of each element, the eight elements at each face, and the six pyramids of the die are connected with dotted lines. They are always interchanging and balancing one another. The solid line outlined the die represents the absolute of health. The absolute of health means that it is not expandable or reducible, the only thing that can be altered is the health status which is implicated by the interaction of Yin and Yang of the body [28]. Specifically in this study, health status is referred as the blood pressure state of a person. 

### 1.2. The Six Locations

The six faces are the six locations (left and right cun, guan, chi) where pulses are assessed by a TCM doctor. The lung and the heart, the liver and the spleen and, the kidney and the kidney (lifegate) are arranged in opposite faces according to the location their health reflected. Such arrangement is based on the fact that left cun, guan, and chi assess blood which is Yin in nature and right cun, guan, and chi assess qi which is Yang in nature in TCM pulse diagnosis [3, 28].

### 1.3. The Eight Elements

Each face is composed of the eight elements. The enlarged face at the lower right shows the interrelation of the eight elements. Each element is a complementary pair of Yin and Yang. According to Yin Yang theory, Yin is always inside and Yang is always outside [28]. So, the black square indicating the Yin nature of the elements is at the core of the face and the white square indicating their Yang nature is at the outer part of the face. The intensity of the eight elements depends on the arterial pulse. The combination of the eight elements indicates the health status of the organs. 

Since this is a dynamic model with many sophisticated nonlinear relationships in between constructs, artificial neural network (ANN) is an ideal modeling technique to model these relationships [29, 30]. Validity of the ANN models was evaluated by its accuracy, sensitivity, and specificity. 

## 2. Materials and Method

This was a cross-sectional study with two parts. Part 1 was the content validation test. Part 2 focused on comparing accuracy, sensitivity, and specificity of the ANN models for differentiating essential hypertension from normotension.

### 2.1. Content Validation

A TCM pulse assessment form was designed to measure the intensity of eight elements at six locations. There were six sections in the form, corresponding to the six locations. In each section, depth, rate, width, length, smoothness, stiffness, and strength were rated using a visual analog scale (VAS).

For depth, the scale ran from “deepest” to “most floating”. Rate was scaled from “slowest” to “fastest”, width from “smallest” to “largest”, length from “shortest” to “longest”, smoothness from “roughest” to “smoothest”, stiffness from “least stiff” to “stiffest”, and strength from “least forceful” to “most forceful”. As length was measured across cun, guan, and chi, it contained only two items-length at the left side and length at the right side. Regularity was a categorical variable that was either regular (0) or irregular (1). 

The content validation was performed by five TCM experts in a local university. They commented on the relevancy of the items and the use of the anchoring words at the ends of the VAS and rated the relevancy of the items on a four-point scale. The four-point scale comprised “irrelevant”, “somewhat relevant”, “relevant”, and “very relevant”. A Content Validation Index (CVI) was calculated as the percentage of agreement over the list of items among the five experts.

### 2.2. Criterion Validation

Data collection took place at a TCM laboratory in the School of Nursing of the university from June to October 2008. Ethical approval was obtained from the Research Committee of the School of Nursing. The laboratory temperature was kept at 22 degrees Celsius throughout the study period, as fluctuations in ambient temperature are known to affect the pulse [7]. 

### 2.3. Selection and Descriptions of Participants

Volunteer subjects were recruited through quota sampling using e-mails, posters displayed on the university campus and in community health centers and hospitals, and newspaper advertisements. Inclusion and exclusion criteria governed the subject selection, as summarized in Table 1, for normotensive and hypertensive groups. 

Subjects eligible for the hypertensive group were required to stop taking antihypertensive drugs one day before their appointment. The purpose, procedure, and potential effects of the study were described and explained to the subjects, who were told that they could withdraw from the study at any time without penalty. Written consent was obtained before the data collection. 

At the beginning of data collection, the subjects were asked to lie on a bed for 20 minutes before data collection, as it has been demonstrated that hemodynamic modification stabilizes after 20 minutes in a new position [32]. After a 20-minute rest, their demographic data, blood pressure, and pulse readings were collected. 

The TCM doctor assessed pulses at left and right cun, guan, and chi and rated the intensity of the eight elements on the validated TCM pulse assessment form while the subject was in a supine position. The whole process took less than 30 minutes. 

Descriptive statistics were computed to determine the distribution of the data with Statistical Package for Social Sciences (SPSS) version 15. Mann Whitney *U* and independent *t*-tests were used to compare mean difference of subjects' clinical data. ANN was used to compute the nonlinear relationship with MatLab 8.0. 

### 2.4. Artificial Neural Network

Three factors were manipulated in the modeling: the number of hidden layers, the number of hidden neurons, and the training algorithms used. As there is no protocol in ANN studies to guide the training process, the three factors were systematically adjusted during the modeling to generate the best results. 

To increase the efficiency of the training, the input data and target data were preprocessed before training commenced. The input and target data were first normalized to a zero mean and a unity standard deviation, and the numbers of neurons in the input and output layers were fixed. The dependent variable was a group (either normotension or hypertension), which was assigned a value of 0 for normotension and 1 for hypertension. The eight elements at the six locations were the independent variables; the input neurons were the eight elements at the six locations, and the output neuron was a group. 

A three-layer ANN was initially used, and the number of hidden neurons was set at 10, and increased at five-neuron intervals until the performance of the model leveled off or decreased. Both backpropagation and radial basis networks were tried during modeling. Bayesian Regularization, Levenberg-Marquardt, and Resilient Backpropagation algorithms were used for the backpropagation. Log-sigmoid function and pure linear function were used as the transfer functions to connect the input layer to the first hidden layer and the hidden layer to the output layer, respectively. If the performance was not satisfactory, then a four-layer ANN was tried. 

A probabilistic neural network is a radial basis network that uses a Bayesian classifier to estimate the probability. There is only one hidden layer in the network, and the radial basis function is used as the transfer function. The transfer function connecting the hidden layer to the output layer is a compete function that chooses the class with the largest probability [33]. 

The performance of the models was evaluated according to their sensitivity, specificity, and predictive accuracy. 

## 3. Results

### 3.1. Content Validation

There were 44 items in the TCM pulse assessment form. Thirty-two items were agreed upon by all five experts, but two experts disagreed on items 3, 7, 10, 14, 17, 21, 25, 29, 32, 36, 39, and 43. “Agreed” refers to a rating of “relevant” or “very relevant” and “Disagreed” to a rating of “irrelevant” or “somewhat relevant.” The two experts that disagreed on the twelve items explained that although there was no standard for assessing pulse qualities in TCM, they did not think that the twelve items were good enough to represent TCM pulse diagnosis. However, as the majority of the experts on the panel agreed on these items, the researcher retained these items on the TCM pulse assessment form. 

To compute the percentage of agreement, the number of “agreed” items was first calculated and then divided by the total number of items retained in the content validity assessment rating form.

(1) $$\begin{array}{cc} & \text{\%}\;\text{of}\;\text{agreement}\;\left(\text{CVI}\right)\\ & =\text{number}\;\text{of}\;\text{agreed}\;\text{items}/\text{total}\;\text{number}\;\text{of}\;\text{items}\;\text{retained}\\ & =\frac{32}{44}=0.73. \end{array}$$

The content validity index was thus 0.73, which is acceptable. 

### 3.2. Criterion Validation

260 subjects were recruited, of which 139 were in the normotensive group and 121 were in the hypertensive group. A Mann Whitney *U*-test was used to examine the differences in gender, age, and body mass index between the groups. The differences in pulse rate, left-side blood pressure, and right-side blood pressure were tested by using an independent *t*-test. The demographic information, blood pressures, body mass index, and pulse rate of the subjects, is presented in Table 2. There was no statistically significant difference in gender, age, or body mass index between the groups (*P* > 0.05), but a statistically significant difference in pulse rate, left-side blood pressure, and right-side blood pressure was found (*P* < 0.01). 

Table 3 compares the results of three back-propagation training algorithms with 10, 15, 20, and 25 hidden neurons. The value reported is the average of 10 trainings. The accuracy of the three algorithms increased slightly with an increasing number of hidden neurons, and ranged between 0.74 and 0.79. Their specificity, however, dropped with an increasing number of hidden neurons, and their sensitivity stopped increasing at 15 hidden neurons.

The specificity, sensitivity, and accuracy of the ANN generated with the probabilistic neural network was 0.68, 0.78, 0.74, respectively (Table 4). Table 4 compares the best model for each ANN algorithm and the model developed by logistic regression. 

A model with a high true positive rate is preferable, which means that the sensitivity should be sufficiently high while the specificity is preserved. The models developed by Bayesian regularization and resilient back-propagation achieved a similar specificity and sensitivity, which establishes that either Bayesian regularization or resilient back-propagation is the best training algorithm for developing the diagnostic model. The results rejected the null hypothesis that the TCM pulse diagnostic model cannot differentiate essential hypertension from normotension. 

## 4. Discussion

This is the first study proposing a model quantifying TCM pulse diagnosis with the eight elements at the six locations. The result of content validation suggests that the model fits into the content of TCM pulse diagnosis. As compared to our study, the one proposed by King et al. [11] did not meet the assessment criteria of TCM pulse diagnosis which is the eight elements at the six locations. Our result is therefore more relevant to TCM pulse diagnosis. 

In our study, VAS was used to measure the intensity of the eight elements. VAS is a method used to assess subjective experience, which in this study was the intensity of the eight elements [34]. A VAS consists of a 10 cm line with both ends marked with anchors. The method is simple to use and its validity and reliability have been established [35]. It is congruent with the usual practice of pulse assessment in TCM and provides various ratings for a TCM doctor to choose that do not mask the actual sensation felt by the doctor. A numerical or ordinal scale would compress the information collected, because the choice would be restricted and the descriptors and numerals anchored to the numerical or ordinal scale could potentially introduce subjective judgment. As a VAS is administered in paper and pen format and requires the researcher to measure a mark made on the line with a ruler, measurement error can occur. However, it has been showed that the VAS has good construct validity in clinical and research use [36, 37] and fits the concept of TCM pulse diagnosis. It was thus used to measure the intensity of the eight elements. 

The results of criterion validation demonstrated that the proposed model is valid enough, as reflected in its accuracy, sensitivity, and specificity, to discern essential hypertension from normotension. The hypothesis set in this study is therefore rejected. Nonetheless, it must also be noted that several hypotheses in the model are yet to be falsified. The basis of TCM pulse diagnosis is Yin Yang theory and the Eight Principles. The bipolar Yin Yang nature of each element has not been examined in this study. As the ultimate purpose of TCM pulse diagnosis is to help in syndrome differentiation which is based on the Eight Principles, the model should therefore comply with the Eight Principles. Chinese medical texts do mention the correspondence of each element with the three bipolar pairs (exterior/interior, heat/cold, excess/deficiency) in the Eight Principles. Depth indicates the interior-exterior pair; rate and regularity indicates the heat-cold pair; width, length, smoothness, stiffness, and strength indicate excess-deficiency pair [7, 14]. But no evidence is so far available to verify these claims. Future studies are recommended to focus on falsifying these hypotheses. In addition, the weight of each element and location on a specific health status is not readily retrieved due to the black-box nature of ANN model [38]. With more advanced and powerful computers, we believe that more information could be retrieved from the model. 

This study has several limitations. The subjects recruited were in a stable condition, and thus the model developed is confined to stable hypertensive cases and cannot be extrapolated to other conditions such as severe hypertension and hypotension as described by National Heart Lung and Blood Institute [31]. It is recommended to recruit subjects with severe hypertension or hypotension in future studies to increase the generalizability of the model. Also, we cannot generalize to other disease differentiation because the results in this study only validated the model with essential hypertension. It is recommended to validate the model by comparing its accuracy with the differentiation of other diseases in future studies. Finally, because only one TCM doctor was recruited to perform pulse diagnosis, individual bias may have affected the results.

## 5. Conclusions

This was the first study presenting a quantifiable TCM pulse diagnostic model with the eight elements at the six locations. The results demonstrated the model's content matched with the context of TCM pulse diagnosis and it attained an acceptable accuracy, sensitivity, and specificity in the context of essential hypertension. Further works are required to verify other hypotheses in the model as pointed out in previous paragraphs. 

## Figures and Tables

**Table 1:** Selection criteria of normotensive and hypertensive subjects.

Selection criteria	Normotension	Hypertension
Inclusion criteria	(i) Aged 18 or above	(i) Aged 18 or above
	(ii) SBP <120 mmHg and DBP <80 mmHg at rest* [31]	(ii) Diagnosis of essential hypertension
		(iii) SBP ≥140 mmHg/DBP ≥90 mmHg or both* [31]
Exclusion criteria	(i) Pregnant	(i) The same as those in normotension except the current use of antihypertensive drugs
	(ii) Loss of upper extremities	
	(iii) Chronic diseases	
	(iv) Infectious diseases	
	(v) Current use of any medication including prescription from a medical doctor, herbal medicine and OTC drugs	

* SBP: systolic blood pressure; DBP: diastolic blood pressure.

**Table 2:** Background information on the subjects (*N* = 260).

		Normotensive group^*ψ*^(*n* = 139)	Hypertensive group^*ψ*^(*n* = 121)	Significance* (*P* < 0.05)
Gender	Male	55	53	—
	Female	84	68	
Age (yrs)	18–34	43	9	—
	35–64	86	93
	≥65	10	19
BMI (kg/m^2^)	<18.50	12	5	—
	18.50–22.99	103	96	
	≥23.00	24	20	
LBP (mmHg)		112 (13)/68 (7)	150 (16)/95 (11)	0.01
RBP (mmHg)		113 (13)/68 (7)	150 (15)/95 (11)	0.01
Pulse Rate (bpm)		65 (10)	70 (11)	0.01

BMI: Body mass index; LBP: Left-side blood pressure; RBP: Right-side blood pressure.

***^*ψ*^***The mean (SD) is reported for the continuous variables and the frequency for the categorical variables.

*P < 0.05 denotes statistical significance.

**Table 3:** Comparison of the specificity, sensitivity, and accuracy of the three back-propagation training algorithms with different numbers of hidden neurons (*N* = 260).

Algorithm	Number of hidden neurons	Specificity (%)	Sensitivity (%)	Accuracy (%)
Bayesian regularization	10	69.96	76.57	73.50
	15	73.46	84.49	79.27
	20	73.20	84.80	79.30
	25	69.33	84.80	77.51

Levenberg-Marquardt Algorithm	10	68.29	86.75	78.06
	15	63.17	90.88	77.83
	20	63.67	88.04	76.56
	25	62.93	87.40	75.87

Resilient backpropagation	10	72.19	83.91	78.41
	15	63.67	91.33	78.28
	20	66.61	91.30	79.67
	25	65.85	90.22	78.74

**Table 4:** Comparison of the specificity, sensitivity, and accuracy of the best results of the models using different ANN training algorithms and the logistic regression.

Algorithm	Specificity (%)	Sensitivity (%)	Accuracy (%)	Remarks
Bayesian regularization	73.46	84.49	79.27	15 hidden neurons
Levenberg-Marquardt	68.29	86.75	78.06	10 hidden neurons
Resilient backpropagation	72.19	83.91	78.41	10 hidden neurons
Probabilistic neural network	68.49	78.32	73.76	—
